# Parental and Educator Perceptions of Implementing Standardized Screenings for Early Detection of Motor Skills in Preschoolers: A Representative Survey

**DOI:** 10.1111/cch.70124

**Published:** 2025-06-23

**Authors:** Barbara Scheiber, Sarah Mildner, Peter Federolf

**Affiliations:** ^1^ Department of Sport Science University of Innsbruck Innsbruck Austria; ^2^ Department of Physiotherapy University of Applied Sciences Tyrol Innsbruck Austria

**Keywords:** child development, educator perception, kindergarten, motor skills, parental perception, screening

## Abstract

**Background:**

Motor skill development in early childhood is essential for children's overall growth, including social participation and academic readiness. Despite the importance of motor skills, Austria's preventive health program for preschoolers currently lacks standardized motor screenings. This study explores the perceptions of parents and kindergarten educators regarding the potential implementation of regular, standardized motor skill screenings in kindergartens across Tyrol.

**Methods:**

In April–May 2024, questionnaires were distributed to 25 kindergartens across Tyrol. Parents and educators responded to structured and open‐ended questions on the perceived importance, feasibility and potential impact of mobility screenings. Quantitative data were analysed descriptively, whereas qualitative data underwent thematic analysis to identify key themes regarding attitudes and practical considerations for implementing mobility screenings.

**Results:**

A total of 892 parents and 19 educator teams responded. Both strongly supported mobility screenings, with 90.6% of the parents viewing them as important as existing health screenings and 100% of educators valuing their importance regarding children's motor development. Qualitative responses from parents emphasized the role of mobility screenings in promoting general development while highlighting the need for a child‐friendly, voluntary approach. Educators not only showed strong support, noting the feasibility and benefit of daily routines, but also identified logistical and communication challenges regarding follow‐up. Both groups acknowledged the potential of standardized screenings for early identification, allowing timely developmental support.

**Conclusion:**

The findings underscore the perceived importance of mobility screening in kindergarten to identify motor skill deficits in early childhood, an insight strongly supported by both educators and parents. Stakeholder support is a critical prerequisite for potential policy adaptations towards offering standardized kindergarten mobility screenings. To ensure effective integration, practical factors such as logistics, tool selection and accessibility must be addressed.

**Summary**

- High parental and educator endorsement supports policy implementation.
- Early screening facilitates timely identification of motor skill deficits.
- Interdisciplinary collaboration is essential for effective delivery.
- Equitable access necessitates addressing linguistic and structural barriers.

## 1 Introduction

Preschool age is a critical period for early childhood development, during which children acquire fundamental motor, cognitive and social skills (Griebler et al. 2013). These early developments form the foundation for various aspects of a child's growth, influencing their participation in different activities throughout childhood. Children with motor impairments participate less frequently and in a narrower range of activities compared to their typically developing peers and may also have a tendency to be overweight (Fong et al. 2011). Motor skills are fundamental for social play, facilitating the development of vital social‐communicative and executive functions. Consequently, the quality of motor skills directly impacts children's ability to adapt to and interact with their environment (Fogel et al. 2023; MacDonald et al. 2017). Limitations in motor skills restrict opportunities for exploration, thereby significantly increasing the risk of broader developmental delays (Lobo et al. 2013).

Like other developed countries, Austria offers a preventive health program for kindergarten children, including an annual examination conducted by a general practitioner or paediatrician, a one‐time ophthalmological examination, a one‐time hearing assessment and a one‐time evaluation of linguistic development (Land Tirol 2024). Those health check‐ups are already successfully integrated into kindergartens. Despite the well‐documented consequences of motor skill difficulties in childhood, such as impaired motor control and limited movement experience in daily life, which can lead to increased insecurity in movement, a higher risk of injury (Kambas et al. 2004), mental health problems (Hill et al. 2016), difficulties in academic achievement (Katagiri et al. 2021), psychosocial maladaptation (Hill et al. 2016; Katagiri et al. 2021) and elevated anxiety levels (Lund Pedersen and Faber Hansen 2022), motor developmental issues have so far received less attention than linguistic development in Austria. Although screenings for the evaluation of children's linguistic development have been conducted in kindergartens for many years, there is no standardized screening for preschoolers' motor skills in Austria (Griebler et al. 2013). Studies have shown that rather subtle developmental deviations, without a clear pathogenesis like developmental coordination disorders (DCD) often go unnoticed, as parents tend to overestimate their children's motor skills (Bolk et al. 2018; Missiuna et al. 2007). Although children with DCD are estimated to represent 5%–6% of the school‐aged population (Blank et al. 2019), this percentage could be even higher due to limited awareness of the condition among parents and healthcare professionals (Al‐Ahmari et al. 2024; Hunt et al. 2021). In a study by Wilson et al. (2013) approximately one‐fifth of the surveyed parents, teachers, family/general practitioners, and paediatricians were familiar with DCD, with 41% of paediatricians and 23% of family/general practitioners reporting awareness of the condition (Wilson et al. 2013). A survey of 150 Austrian kindergarten educators found that 80.6% see themselves as responsible for the early detection of motor deficits, whereas 71.5% considered parents and 70.4% paediatricians responsible, with open‐ended responses emphasizing shared responsibility among all stakeholders (Scheiber et al. 2022). These findings highlight critical gaps in the understanding of motor development difficulties among medical professionals, teachers, educators and parents, underscoring the need for more comprehensive initiatives. One approach to addressing this issue is the implementation of a standardized and validated mobility screening in kindergartens, which could help to identify motor difficulties at an early age (Griebler et al. 2013; Griebler and Nowotny 2015; Missiuna et al. 2007). Early identification enables timely referral to a physician for further assessment and diagnosis, allowing for the earliest possible intervention to support optimal development. As we know from linguistic screenings, such assessments offer both disadvantages, including stigmatization, pressure and prejudice, as well as potential advantages, such as early identification and intervention (Eckhardt et al. 2011). Moreover, implementing a screening provides an opportunity to educate parents about motor difficulties in children and raise awareness, which can contribute as a preventive measure against stigmatization and prejudice. To achieve this, it is crucial that parents have a positive attitude towards such screenings, to engage them as relevant stakeholders.

This study is part of a bigger research project (Scheiber et al. 2024) and aims to explore parental and educator perceptions towards a potential implementation of regular and standardized screening of motor skills to assess children's motor development in kindergartens. By compiling arguments for and against such screenings put forward by both stakeholder groups, the study addresses the critical role of parents and educators in determining acceptance and the feasibility of early motor development assessments in early childhood education settings.

## 2 Methods

### 2.1 Study Design

This study was conducted using a cross‐sectional, representative questionnaire survey to explore parental and educator perceptions regarding the potential implementation of regular and standardized motor skill screenings in Tyrolean kindergartens. The study was reviewed and approved by the ethical review boards of both involved universities (certificate 21/2024) and (RCSEQ 3369/24).

### 2.2 Patient and Public Involvement

Relevant stakeholders such as kindergarten educators and parents/legal guardians of 4‐ to 6‐year‐olds were involved in the planning of this study.

### 2.3 Procedure

The questionnaires for parents and kindergarten educators were distributed through the kindergarten management of 25 kindergartens across Tyrol, ensuring a representative distribution with regard to districts as well as urban and rural areas.

### 2.4 Participants

To achieve a representative sample, invitations to participate were sent out in April 2024 to all Tyrolian kindergartens. Seventy‐two out of a total of 486 kindergartens (STATISTIK AUSTRIA 2023) responded positively to the invitation, out of which a sample size of 25 kindergartens was selected based on the population distribution across the nine districts of Tyrol. In each district, the kindergartens with the largest number of children were selected for participation in the study. Regarding the rural–urban distribution, a ratio of 72% (*n* = 18) to 28% (*n* = 7) can be seen within the 25 participating kindergartens. In May 2024, the parental questionnaires were distributed to kindergarten management, who then forwarded them to the parents. The questionnaires aimed at assessing the educators' perceptions were given to kindergarten management directly after the mobility screening had taken place in the respective kindergarten in October 2024. They were asked to complete the questionnaires in the team in a quiet atmosphere.

### 2.5 Response Rates

The parental response rate in urban kindergartens was 62.2% (*n* = 179/288) and 67.8% (*n* = 713/1052) in rural kindergartens, resulting in a total response rate of 66.6% (*n* = 892/1340). The response rate of the kindergarten educator teams was 76% (*n* = 19/25).

### 2.6 Data Collection and Analysis

The data were collected using two semistructured questionnaires designed to assess parental and educator perceptions regarding standardized mobility screenings (see Supplementary Appendices S1 and S2). The questionnaires were developed based on a review of existing literature and underwent a validation process with a pilot study involving 39 participants, which was subsequently revised (Scheiber et al. 2022). The parental questionnaire consists of 10 items, including closed questions on demographic information and questions on a Likert scale that assess the importance of mobility screenings and examinations. In addition, open‐ended questions were used to understand parents' attitudes towards an annual offer of mobility screening and to enumerate any previous motor abnormalities. The educators' questionnaire comprises eight questions, including closed items on demographic and kindergarten‐specific information as well as questions about the level of effort involved in organizing the screening process within the kindergarten. Using a Likert scale, educators were asked directly whether they supported or opposed the implementation of standardized screening in kindergartens and were asked to elaborate on their opinion.

Quantitative data were analysed descriptively using the statistical software Jamovi (jamovi 2024). To capture this complexity, an analytic interpretive orientation was adopted, aiming to remain closely aligned with the participants' voices in the collected data by applying the *hermeneutics of empathy* as described by Braun and Clarke (2021). Qualitative data, including open‐ended responses from the questionnaires, were analysed inductively and data‐driven using thematic analysis, adhering to the iterative six‐step process (Braun and Clarke 2006).

First, the researchers familiarized themselves with the data by reading it repeatedly. In the second step, initial codes were developed to provide a first overview of the data, and intercoder reliability was ensured by two members of the research team, who compared, discussed and reached a consensus on their findings. The codes developed by consensus were applied to the entire dataset. In the third step, the codes were grouped into themes based on their meanings. In the fourth step, the data were revisited with consideration of these themes and adjusted in cases of discrepancies to ensure a thorough analysis. Once the themes were finalized, they were named and defined in the fifth step, enabling the development of a comprehensive narrative. The final phase involved the production of a coherent and detailed report outlining the results of the analysis supported by meaningful examples (Braun and Clarke 2006). Original data analysed for this publication has been made available in a data repository. Reporting of this study adheres to the Standards for Reporting Qualitative Research (O'Brien et al. 2014).

## 3 Results

### 3.1 Descriptive Characteristics

A total of 892 parents of kindergarten children aged 4–6 years participated in the survey. The gender distribution of the children was well‐balanced (50.1% female and 49.9% male). Nineteen out of 25 kindergarten educator teams gave insights into their perception of a standardized mobility screening; 21.1% (*n* = 4) came from urban, and 78.9% (*n* = 15), from rural–urban kindergartens.

Parents expressed a strong appreciation for health screenings for their children, with high importance given to general medical screenings (86.4% rated them as very or rather important), dental screenings (93.9% very or rather important), motor screenings (88.0% very or rather important), visual screenings (93.0% very or rather important) and speech screenings (90.6% very or rather important). Only a small fraction of parents considered these screenings unimportant, with percentages ranging from 0.8% to 2.2%.

Notably, although a standardized mobility screening is not yet part of regular healthcare, parents rated its importance on par with existing screenings; 90.6% (*n* = 778) supported its introduction in kindergartens, suggesting that parents recognize its potential value as highly as that of the already established screenings. Only 9.4% (*n* = 81) expressed opposition to this screening initiative.

Kindergarten educators expressed support for integrating a standardized mobility screening. The question of endorsement or rejection was analysed across three dimensions. First, regarding the significance of motor skills for children's development, all educator teams (*n* = 19) strongly favored introducing the screening. Second, in terms of feasibility within the kindergarten setting, 88.3% supported implementation, whereas 11.7% were rather opposed. Lastly, considering their perception of the warranty of implementing standardized mobility screenings in kindergartens, 88.3% were in favor of the integration and 11.7% were against it.

### 3.2 Qualitative Characteristics

**FIGURE 1:**
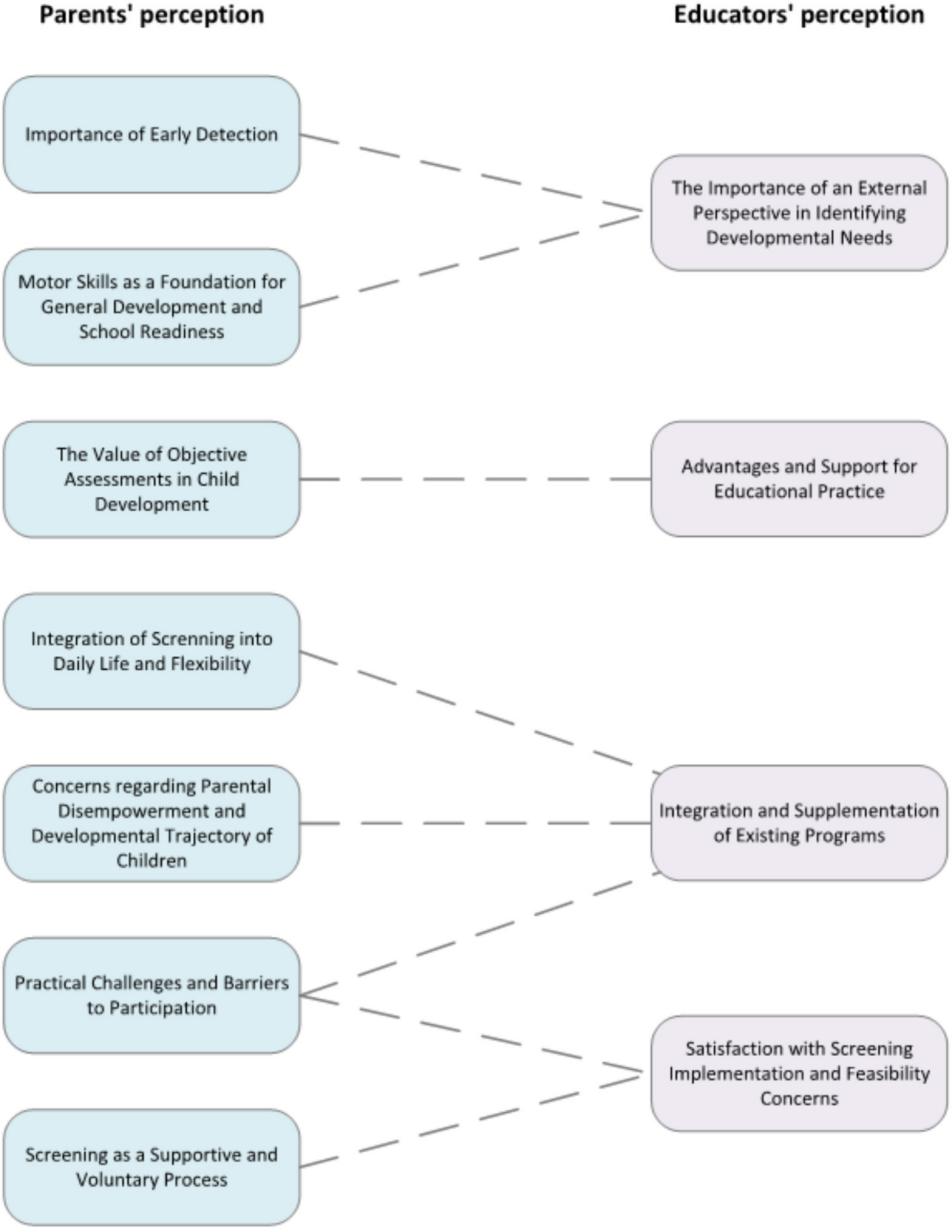
Overview of developed themes regarding parental and educator perceptions and their interrelationship.

The qualitative analysis revealed a range of themes reflecting parents' and educators' perceptions and the perceived feasibility of motor skill screenings in Tyrolean kindergartens. Figure 1 provides an overview of the developed themes and their interrelationships.

#### 3.2.1 Parents' Perception

A total of 518 open responses were evaluated, and as previously mentioned, 90.6% support the implementation of mobility screenings in early childhood education settings. This is why justifications for introducing mobility screenings were mentioned considerably more often than reasons against their implementation. Regarding parents' perceptions, seven themes were developed (see Figure 1).

##### 3.2.1.1 Importance of Early Detection

The most prevalent theme was the early identification of motor skill deficiencies to prevent long‐term developmental issues. One participant emphasized, ‘The earlier something is noticed, the quicker one can react. Parents sometimes become “blind” to the abilities their children should have. Outsiders see a child from a different perspective’. This perspective highlights the necessity of screenings conducted by external experts rather than a continuous observation by people in the daily environment of the child. Other participants stated, ‘Early intervention is important, and we are grateful for the professional assessment’, and ‘The development of our children is very important to us and we provide them with comprehensive support. Early detection of possible impairments or deviations is always an advantage as it increases and therefore improves the chances of successful treatment’. Parents describe that they would like to have this information in order to be able to react: ‘So that I can see whether my child is developing according to age and so that I can react to any abnormalities’. They recognized early identification as an opportunity and early intervention as a necessary consequence to prevent subsequent challenges. Trust in external expertise strengthened the belief that these evaluations could lead to timely and effective support.

##### 3.2.1.2 The Value of Objective Assessments in Child Development

The role of professional, external assessments was deemed highly valuable. As noted by a participant, ‘You receive a professional assessment in a very simple way. In this setting, there is much more time for detailed observations than during the paediatrician's routine child health examinations’.^1^ The objectivity and impartiality of these assessments were viewed as critical to ensuring accurate and unbiased interventions. One parent highlighted the challenge of assessing their child's motor development, noting a ‘lack of opportunities for comparison—what is “normal”, what is not? Especially when observing movement sequences that are difficult to interpret, such as swimming, pedaling, or climbing lessons’. This suggests that parents often struggle to determine on their own whether their child is experiencing motor difficulties. Physiotherapists, in this context, are regarded as movement experts whose external perspective is valued: ‘The view from the outside, from an expert, is to be welcomed’. Additionally, feedback from independent professionals appears to strengthen parental confidence: ‘It would be great if parents could get feedback from “strangers”.’

##### 3.2.1.3 Motor Skills as a Foundation for General Development and School Readiness

Motor skill development was perceived as fundamental for children's overall growth, influencing both health and cognitive outcomes. This theme reflects the recognition that motor skills play a crucial role in school readiness and long‐term career success. One participant stated, ‘I find it important to screen motor skills before school entry, as they are, for me—just like language skills—fundamental building blocks for academic success. Mobility screenings should therefore become standard in kindergartens’.

Participants further acknowledged that the development of motor skills is integral to various aspects of life. As one participant articulated, ‘Health, prevention, and future actions. Life planning, education, and care are all dependent on these [motor skills]’. This statement highlights the recognition of the interdependence of motor skills with health and educational outcomes.

##### 3.2.1.4 Screening as a Supportive and Voluntary Process

This theme emphasizes the role of screenings as a supportive process rather than merely a measurement tool. Participants highlighted the importance of voluntariness and autonomy, allowing families to choose freely whether to participate in assessments without coercion. One participant articulated, ‘Depending on whether it makes sense and if there is also a follow‐up low‐threshold support program. No, if it remains only at the level of “evaluation”’. This statement underscores the necessity for screenings to be part of a comprehensive support system that extends beyond mere evaluation.

Preventive screenings were viewed as a standard practice, akin to other health checks, reinforcing their routine nature and significance in child development. As one participant noted, ‘Since I generally take my children to preventive check‐ups (dentist, pediatrician, eye doctor), a mobility assessment by professionals is also a meaningful addition’. This perspective highlights the alignment of motor skill assessments with other established health practices, further supporting the case for their routine implementation in early childhood settings.

##### 3.2.1.5 Concerns Regarding Parental Disempowerment and Developmental Trajectory of Children

This theme addresses the emotional distress experienced by parents regarding external assessments of their child's development. Participants expressed feelings of insecurity and disempowerment, perceiving their autonomy in evaluating their child's progress as compromised. One participant stated, ‘I wouldn't seek an external assessment, as I believe I can evaluate this myself, but if it's offered without any effort on my part, I find it positive’. This highlights the ambivalence parents feel towards unsolicited evaluations; although they appreciate convenience, they desire control over their child's assessment. Parents also acknowledged the complexity and variability of child development, recognizing the need for individualized approaches rather than standardized screenings. One participant noted, ‘A child's development is difficult to generalize/systematize, and the assessment of abilities can also create pressure for both parents (and the child). Every child develops at their own pace’. This underscores the challenges of using standardized assessments to capture the uniqueness of each child's development. Scepticism about the necessity of these assessments was evident, with one participant stating, ‘Every child develops at their own speed. It's just another ‘measurement’ to unsettle parents’. Another remarked; ‘I don't see the need; routine health check‐ups… are sufficient’. From the parental perspective, these sentiments suggest that existing health assessments are perceived as sufficient for addressing developmental concerns, with no perceived need for additional screenings. The feelings of disempowerment and stress, along with the complexities of child development, underscore the need for sensitivity and support in involving parents throughout the assessment process. Addressing parental concerns can foster a collaborative environment that empowers families and enhances their engagement in their child's development.

##### 3.2.1.6 Integration of Screening Into Daily Life and Flexibility

This adaptability and the ability to incorporate assessments in a playful, child‐friendly manner were viewed as vital for encouraging participation without disrupting daily activities. The practical integration of motor skill assessments into children's daily routines was perceived as essential for ensuring that screenings were unobtrusive and smoothly incorporated into educational environments. Parents recognized the increasing importance of motor skills due to changes in children's lifestyles, such as reduced physical activity. One participant stated, ‘I support low‐threshold screenings in the children's familiar environment and prefer these over quickly handled parents‐child health passport medical examinations’. This preference highlights the need for assessments to be conducted in a setting that feels comfortable and familiar to children, thereby facilitating a more effective screening process. The statement, ‘As long as it happens in a playful manner and no pressure to perform is conveyed’ emphasizes the importance of creating a nonthreatening environment that fosters engagement.

Participants also acknowledged the rising prevalence of motor skill issues among children, attributing this trend to lifestyle changes. One participant remarked, ‘Motor skill issues in children are unfortunately becoming more common nowadays (lack of movement, tablets, smartphones, etc.). Fine motor development and language development are closely connected’. This statement underscores the urgent need to raise awareness of the significance of motor skills, both independently and in connection with other areas of development.

##### 3.2.1.7 Practical Challenges and Barriers to Participation

This theme encompasses the logistical and communicative barriers faced by both parents and children. Parents with children already exhibiting developmental concerns felt overburdened by numerous appointments and obligations. One participant expressed, ‘That would be too much for my child, as they regularly attend appointments in the capital city about 3–4 times a year and go to occupational therapy locally. The issues are already identified and being treated’. This statement highlights the strain on families who are already managing multiple commitments, making it challenging to add additional screenings to their schedules. Ensuring children's cooperation during screenings, coupled with language barriers for nonnative speakers, further complicated participation. As one participant noted, ‘We have different mother tongues, so it's hard to speak in German’. This indicates that linguistic differences can hinder effective communication during screenings, potentially impacting the children's engagement and therefore the screening result.

#### 3.2.2 Educators' Perception

The qualitative analysis revealed four themes, evaluating 17 open answers, in relation to educators' perceptions of mobility screenings in kindergartens (*see* Figure 1).

##### 3.2.2.1 The Importance of an External Perspective in Identifying Developmental Needs

The screening is perceived as essential by educators, who find it beneficial for both their professional practice and for parents. The respondents emphasize the importance of the observations made during the screening, noting that ‘For us educators, the observations during the screening are very interesting and helpful’. Furthermore, they highlight the significance of professional assessments, particularly in the context of identified deficits, with one participant stating, ‘For parents, an assessment in this area is often important, especially when a therapist identifies “deficits”’.

##### 3.2.2.2 Advantages and Support for Educational Practice

Participants believe that the screening offers significant advantages for educational practice by providing useful insights that can be incorporated into developmental discussions. However, concerns about a lack of feedback are noted, with educators expressing a desire for information that could enhance their work, ‘For us, there is no information, even though the expertise could support us in our daily work’. The potential for improved communication of identified abnormalities to the kindergarten staff is also highlighted, with a participant stating, ‘It would be desirable if any abnormalities could be communicated to the kindergarten in order to establish targeted offerings and support opportunities’. The educators expressed interest in discussing the results with the physiotherapists, stating, ‘As educators, we are always very happy to engage in an exchange of opinions’.

##### 3.2.2.3 Satisfaction With Screening Implementation and Feasibility Concerns

The respondents consistently report positive experiences regarding the implementation of the specific screening that took place. They describe the organization, communication and implementation as ‘uncomplicated’ and ‘well‐organized’, indicating a high level of satisfaction with the process. However, one participant expressed concerns about the practical feasibility of implementing the screening within the daily routines of kindergartens, with one comment highlighting potential issues, ‘I think the feasibility in the kindergarten context could be a challenge’.

##### 3.2.2.4 Integration and Supplementation of Existing Programs

The motor screening is viewed as a valuable addition to the existing kindergarten preventive care program, which is reported to function effectively. Respondents believe that it can be seamlessly integrated into the daily practices of kindergartens, with one stating, ‘The existing kindergarten preventive care program works very well and is uncomplicated, so a mobility screening could also be implemented in the kindergarten routine’. This would eliminate the barriers to implementation mentioned by the educators, which were attributed to the study's administration and the associated administrative burden on parents, as one educator's statement illustrates, ‘The registration form for parents is very complicated. Many details need to be filled out twice—an additional checkbox for the routine screenings would be great’. Another suggestion from educators, ‘We could have possibly prepared the weighing and measuring of the children ourselves’, would no longer be specifically necessary for the motor skills screening, but rather could be covered within the framework of the existing routine procedures.

## 4 Discussion

This study explored parental and educator perceptions of standardized motor skill screenings in Tyrolean kindergartens, where such screenings are not yet routinely implemented, unlike linguistic screenings that have been conducted by speech therapists for many years (Land Tirol 2024). The results provide substantial insights into both groups' attitudes towards mobility screenings, reflecting high levels of support while also revealing several concerns.

The results of this survey show that the introduction of standardized mobility screenings in Tyrolean kindergartens is strongly supported by both parents and kindergarten educators. The considerable number of open comments from parents, as well as the contributions from kindergarten teachers, emphasize the relevance of this topic in early childhood education. The responses show that the value of screenings for early detection of motor deficits, supporting overall child development and promoting school readiness is widely recognized, which is very positive, as existing studies show that the awareness of certain deviations is very low among both health professionals and primary caregivers. Previous scientific literature reports that parents often experience motor difficulties as ‘clumsiness’ that kids would grow out of (Hunt et al. 2021; Steenbergen et al. 2024). A study from 2021 highlighted that parents often lack information about motor development, leading them to believe that children develop even complex motor skills naturally, which results in a diminished perception of the need for support (Agard et al. 2021). This highlights the importance of not only providing information but also offering concrete, accessible forms of support once awareness is raised. In this context, parents indicated that, ideally, a low‐threshold follow‐up support program should be implemented following screening to increase acceptance, with options for timely interventions without long waiting times (Eckhardt et al. 2011; Griebler and Nowotny 2015) or counselling sessions for parents provided by physiotherapists. A successful example of such an intervention was demonstrated by Klein et al. (2010), who examined parental education for children with elevated BMI. The study found that even brief counselling sessions can lead to a reduction in BMI (Popovic et al. 2021). Additional approaches to a low‐threshold follow‐up support program could include the integration of play‐based motor development activities in kindergarten settings to enhance endurance (Popovic et al. 2021) and fundamental motor skills (Chen et al. 2024).

The results of this study further show a broad appreciation of existing health screenings by parents, with over 85% categorizing medical, dental, visual and linguistic screenings as important for their children. The finding that 90.6% of parents favored the inclusion of mobility screenings on an equal footing with these established screenings is remarkable given that this is not yet a routine part of health screenings. This suggests that parents welcome a more comprehensive monitoring of their children's development. The emphasis on early identification as a means of preventing long‐term developmental problems emerged as a key theme, reflecting parents' recognition of the role that external, professional assessments can play in identifying deficits that may go unnoticed during day‐to‐day observation by family members.

Early recognition of abnormalities in motor development is crucial in preventing persistent and related effects, such as anxiety, depression (Draghi et al. 2021), difficulties in handwriting (Dionne et al. 2023; Rosenblum et al. 2013), mathematics (Gomez et al. 2017; Pieters et al. 2012) and social skills (Tal‐Saban et al. 2021). The strong parental support was echoed by kindergarten teachers, with 92.2% being in favour of carrying out mobility screenings, highlighting their feasibility and usefulness in educational institutions. Educators emphasized their importance not only for identifying motor problems but also for enriching their professional practice. Integrating motor screenings into existing kindergarten routines was seen as meaningful and feasible, although one educator expressed concerns about the potential disruption of daily activities. Evidence also supports the successful implementation of not only screenings but whole movement (training) programs in kindergartens (Vidoni et al. 2014). Our results showed that parents associated motor skills with a wide range of developmental outcomes, including cognitive performance and school readiness, which is well supported by existing literature linking motor skills to school readiness (Aydoner and Bumin 2023; Jones et al. 2021).

The feasibility of the practical integration of such a screening into the kindergarten routine was seen as essential, with parents emphasizing the need for child‐centred, low‐stress approaches to ensure participation without placing undue pressure on children. Despite the strong support for mobility screenings, several barriers to their implementation were identified. Educators highlighted practical challenges, such as the administrative burden of registration forms, which was greater than for other established screenings—this was primarily due to the specific requirements of a scientific study setup. Educators highlighted the need for interprofessional cooperation between health professionals and educators after testing. This commitment to interprofessional collaboration is both valued and reinforced by existing literature, particularly regarding the partnership between speech therapists and educators (Glover et al. 2015). Additionally, parents with children who already attend multiple health appointments, particularly in rural areas, expressed concerns about the additional burden of another obligation. Language barriers also emerged as a potential obstacle, particularly for nonnative speakers, which could hinder communication during screenings. Furthermore, some parents expressed skepticism about the need for standardized screening, citing the uniqueness of each child's development and the potential for screening to cause unnecessary stress for families. In this context, research indicates that parents often overestimate their children's abilities, highlighting the necessity for screenings conducted by movement specialists (Deimann and Kastner‐Koller 2011; Zysset et al. 2018).

Another criticism of preschool screening is the increased pressure on children to perform (Eckhardt et al. 2011). Furthermore, parents express concerns about self‐ or external stigmatization due to labeling, prejudice or social exclusion, which have also been highlighted in previous studies (Griebler and Nowotny 2015). Diagnostic labeling in general can have both positive and negative psychological effects on individuals. Literature assumes that nearly 72% of studies about diagnostic labeling reported negative outcomes, including resistance, shock, anxiety, confusion, feelings of loss, abandonment, fear, sadness and anger. Conversely, 61% of studies indicated that a diagnostic label could have a positive impact, with individuals often feeling relief, validation, legitimization and empowerment. Other reported benefits included increased hope, reduced uncertainty, better communication with others and improved self‐understanding (Sims et al. 2021). Screenings can also raise awareness of motor problems in childhood, which can reduce stigma. Nevertheless, these concerns suggest that the introduction and implementation of the screening must be well integrated into the kindergarten environment and handled in an informed manner to ensure it is supportive and beneficial, without appearing intrusive. At the same time, the selection of an appropriate screening tool remains a major challenge, particularly as existing questionnaires and short motor tests often lack sufficient accuracy for broad population screening (Cools et al. 2009; Zysset et al. 2018).

All themes were generated through a reflexive and collaborative process. This process was inherently shaped by personal experiences and expectations, influenced by the shared background of both researchers as physiotherapists. Although this perspective enriched our analysis, it may also represent a limitation of the qualitative findings, as it could have introduced a degree of interpretive bias.

## 5 Conclusion

The results of this study provide valuable pointers for policy decisions indicating strong support for the introduction of standardized mobility screenings in Tyrolean kindergartens. Both parents and educators recognize the value of early identification of motor deficits as a means of promoting more comprehensive developmental outcomes. However, practical considerations, including logistical challenges, selection of screening tools and language barriers, should be considered to ensure the successful implementation of such screenings. Future policy efforts should focus on integrating these screenings into the kindergarten routine in such a way that mobility screenings can be effectively incorporated into early childhood education for the benefit of all stakeholders.

## Author Contributions

**Barbara Scheiber:** conceptualization, investigation, funding acquisition, writing – original draft, methodology, validation, visualization, formal analysis, project administration, data curation. **Sarah Mildner:** investigation, writing – original draft, methodology, validation, formal analysis, data curation. **Peter Federolf:** conceptualization, writing – review and editing, supervision.

## Ethics Statement

Research Committee for Scientific Ethical Questions (RCSEQ 3369/24) and Ethical Board of the University of Innsbruck (certificate 21/2024).

## Consent

The authors have nothing to report.

## Conflicts of Interest

The authors declare no conflicts of interest.

## Supporting information


**Data S1.** Parental Questionnaire.

**Data S2.** Educator Questionnaire.

## Data Availability

Original data analysed for this publication has been made available in a data repository (10.6084/m9.figshare.27722133.v1). Informed consent forms for the kindergartens and parents as well as information sheets in German language are available on request.

## Endnotes

^1^
The parent–child passport is an Austrian health document that provides essential preventive examinations (parent–child passport examinations), vaccinations and consultations for the health and development of expectant parents and their children from pregnancy until the age of 3 (parent–child passport [oesterreich.gv.at]).
