# Supplementary material for: Parental and Educator Perceptions of Implementing Standardized Screenings for Early Detection of Motor Skills in Preschoolers: A Representative Survey
Source: Child Care Health Dev. 2025 Jun 23;51(4):e70124. doi: 10.1111/cch.70124 (PMC12186017; doi:10.1111/cch.70124)
Supplement: Supplementary file 1 — Data S1. Parental Questionnaire. [file CCH-51-e70124-s002.pdf]

fhg universität innsbruck

Elternfragebogen

1. Vorname, Nachname Ihres Kindes:

   ____

2. Geburtsdatum Ihres Kindes:

   ____
   (TT.MM.JJJJ)

3. Geschlecht des Kindes:

   ☐ weiblich ☐ männlich ☐ keine Angabe ☐____________________

4. Seit wann besucht Ihr Kind den Kindergarten?

   ____
   (Monat Jahr - Beispiel: September 2021)

5. Kann Ihr Kind Anweisungen in deutscher Sprache verstehen und umsetzen?

   ☐ Ja ☐ Nein

6. Leidet Ihr Kind unter einer angeborenen oder erworbenen Beeinträchtigung?

   ☐ Ja ☐ Nein

   Wenn ja, an welcher?

   ____

7. In Bezug auf Ihr Kind: Wie wichtig ist Ihnen…

0 = gar nicht wichtig, 5 = sehr wichtig

	**0**	**1**	**2**	**3**	**4**	**5**
die jährliche allgemeinmedizinische Reihenuntersuchung?	☐	☐	☐	☐	☐	☐
die Beurteilung der Zahngesundheit?	☐	☐	☐	☐	☐	☐
die Beurteilung der motorischen Fähigkeiten?	☐	☐	☐	☐	☐	☐
die augenärztliche Beurteilung?	☐	☐	☐	☐	☐	☐
die Beurteilung der sprachlichen Fähigkeiten?	☐	☐	☐	☐	☐	☐

Projektverantwortung: Barbara Scheiber, MSc BSc, fhg – Zentrum für Gesundheitsberufe Tirol GmbH, Innrain 98, 6020 Innsbruck, T +43 512 5322-76 763,
barbara.scheiber@fhg-tirol.ac.at, www.fhg-tirol.ac.at

8. Würden Sie ein jährliches Angebot eines Mobilitätsscreenings (Einschätzung der motorischen Fähigkeiten) Ihres Kindes im Kindergarten befürworten?

☐ Ja ☐ Nein

Können Sie Ihre Einstellung bitte in wenigen Worten begründen?

____

____

____

____

9. Haben Sie das Angebot der Mutter-Kind-Pass-Untersuchungen bisher vollständig wahrgenommen?

☐ Ja ☐ Nein

Wenn nein, wie lange haben Sie die Mutter-Kind-Pass-Untersuchungen wahrgenommen? Bis zum…

☐ 1. Lebensjahr ☐ 2. Lebensjahr ☐ 3. Lebensjahr ☐ 4. Lebensjahr ☐ 5. Lebensjahr

10. Wurde bei Ihrem Kind jemals eine motorische Auffälligkeit diagnostiziert oder im Rahmen einer Routineuntersuchung festgestellt?

☐ Ja ☐ Nein

Wenn ja, was wurde festgestellt?

____

Wenn ja, ist diese Auffälligkeit aktuell noch bestehend?

☐ Ja ☐ Nein

Wir bedanken uns herzlich für Ihre Bereitschaft diesen Fragebogen auszufüllen und Ihrem Kind eine Teilnahme am Mobilitätsscreening zu ermöglichen.

Bitte geben Sie <u>diesen ausgefüllten Fragebogen gemeinsam mit der unterschriebenen Einwilligungserklärung</u> im beiliegenden Umschlag bis 29. Mai 2024 bei der:dem Kindergartenpädagog:in ab.

Projektverantwortung: Barbara Scheiber, MSc BSc, fhg – Zentrum für Gesundheitsberufe Tirol GmbH, Innrain 98, 6020 Innsbruck, T +43 512 5322-76 763, barbara.scheiber@fhg-tirol.ac.at, www.fhg-tirol.ac.at
