# Supplementary material for: Parental and Educator Perceptions of Implementing Standardized Screenings for Early Detection of Motor Skills in Preschoolers: A Representative Survey
Source: Child Care Health Dev. 2025 Jun 23;51(4):e70124. doi: 10.1111/cch.70124 (PMC12186017; doi:10.1111/cch.70124)
Supplement: Supplementary file 2 — Data S2. Educator Questionnaire. [file CCH-51-e70124-s001.pdf]

Reflexionsfragen an die Kindergartenleitung

1. Lage des Kindergartens

   ☐ ländlich ☐ städtisch

2. Handelt es sich bei dem Kindergarten, in dem Sie tätig sind, um eine besondere Form eines Kindergartens, wie beispielsweise einen Waldkindergarten oder einen Montessori-Kindergarten?

   ☐ nein, es handelt sich um einen Regelkindergarten

   ☐ ja: unser Kindergarten ist ein ______________________________

3. Wie groß ist Ihr Kindergarten?

   Anzahl der Gruppen:________________bzw. Anzahl der Kinder: ________________

   Anzahl der Pädagog:innen ___________bzw. Assistent:innen:__________________

4. Wie schätzen Sie den Aufwand ein, der sich für Sie durch die Teilnahme an diesem Projekt ergab?

0 = sehr niedrig, 5 = sehr hoch

	0	1	2	3	4	5
Kommunikation mit dem Projektteam	☐	☐	☐	☐	☐	☐
Terminvereinbarungen mit dem Projektteam	☐	☐	☐	☐	☐	☐
Kommunikation mit den Eltern	☐	☐	☐	☐	☐	☐
Planung und Organisation des Screeningablaufs von Ihrer Seite	☐	☐	☐	☐	☐	☐
Aushändigen und Einsammeln der Dokumente	☐	☐	☐	☐	☐	☐
Allgemeine Einschätzung des Gesamtaufwandes	☐	☐	☐	☐	☐	☐

Projektverantwortung: Barbara Scheiber, MSc BSc, fhg – Zentrum für Gesundheitsberufe Tirol GmbH, Innrain 98, 6020 Innsbruck, T +43 512 5322-76 763,
barbara.scheiber@fhg-tirol.ac.at, www.fhg-tirol.ac.at

5. Haben Sie Vorschläge für eine künftige Verbesserung des Ablaufes eines solchen Screenings?

____

____

____

____

____

6. Gibt es Aspekte, wo Sie sich mehr Unterstützung von unserer Seite gewünscht hätten?

   ☐ Ja ☐ Nein

   Können Sie dies bitte in wenigen Worten erläutern?

____

____

____

____

____

7. Gibt es Aspekte, wo Sie sich mehr Einbindung in das Screening gewünscht hätten?

   ☐ Ja ☐ Nein

   Können Sie dies bitte in wenigen Worten erläutern?

____

____

____

____

____

Projektverantwortung: Barbara Scheiber, MSc BSc, fhg – Zentrum für Gesundheitsberufe Tirol GmbH, Innrain 98, 6020 Innsbruck, T +43 512 5322-76 763, barbara.scheiber@fhg-tirol.ac.at, www.fhg-tirol.ac.at

8. Wie stehen Sie dem Vorschlag gegenüber, ein Mobilitätsscreening durch Physiotherapeut:innen in das Kindergarten-Vorsorgeprogramm des Landes Tirol zu integrieren?

0 = starke Ablehnung, 5 = starke Befürwortung

	0	1	2	3	4	5
hinsichtlich der Bedeutung der motorischen Fähigkeiten für die Entwicklung eines Kindes	☐	☐	☐	☐	☐	☐
hinsichtlich der Umsetzbarkeit im Kindergartenalltag	☐	☐	☐	☐	☐	☐
hinsichtlich der Notwendigkeit vor dem Hintergrund der aktuell stattfindenden Routineuntersuchungen und Beobachtungen (im Sinne vom Schließen einer Lücke im Bemühen um Früherkennung und Vorbeugung)	☐	☐	☐	☐	☐	☐

Können Sie Ihre Einschätzungen bitte in wenigen Worten erläutern?

____

____

____

____

____

Wir bedanken uns herzlich für Ihre Bereitschaft diesen Fragebogen auszufüllen und für Ihre Mithilfe und Unterstützung an diesem Projekt!

Projektverantwortung: Barbara Scheiber, MSc BSc, fhg – Zentrum für Gesundheitsberufe Tirol GmbH, Innrain 98, 6020 Innsbruck, T +43 512 5322-76 763, barbara.scheiber@fhg-tirol.ac.at, www.fhg-tirol.ac.at
